# Radiographic manifestations of pneumatosis intestinalis and construction of an AI-assisted diagnostic system

**DOI:** 10.1063/5.0351586

**Published:** 2026-11-02

**Authors:** Xiaodi Zhang

**Affiliations:** General Surgery, Shaanxi Nuclear Industry 215 Hospital, Xianyang 712000, Shaanxi, China

## Abstract

Pneumatosis intestinalis (PI) has a wide range of etiologies, and the imaging differentiation between benign and ischemic PI is difficult, leading to a high risk of missed diagnosis. This study systematically summarizes the CT (Computed Tomography) imaging manifestations of PI and constructs an AI (Artificial Intelligence)-assisted diagnostic model that integrates multidimensional features. This study retrospectively collected CT and x-ray data from 612 patients with PI (398 benign and 214 ischemic cases) from three tertiary hospitals, and included an external validation set of 106 cases. Imaging analysis established a five-level PI-CT severity scoring system based on gas morphology, distribution, portal venous gas (PVG), intestinal wall enhancement, mesenteric edema, and ascites. The AI system adopted a two-level architecture: The first level used EfficientNet-B3 for rapid screening on x-ray films; the second level used 3D nnU-Net (No-new-Net) to segment intestinal wall gas, and then fused the data through a three-branch network (gas morphology, accompanying signs, and clinical metadata) via cross-attention to output the probability of ischemic PI. In the internal test set, the model achieved an AUC (Area Under the Curve) of 0.947 for ischemic PI (sensitivity 91.3%, specificity 93.6%), significantly outperforming senior radiologists (AUC = 0.876); the AUC on the external validation set was 0.931. SHapley Additive exPlanations analysis showed that PVG and blood lactate were the most important predictors. This two-tiered AI system can significantly improve the early identification of the causes of critical PI, providing a reliable method for intelligent diagnosis of emergency imaging.

## I. INTRODUCTION

Pneumatosis intestinalis (PI) refers to an imaging phenomenon in which gas accumulates abnormally in the submucosal or serosal layer of the intestinal wall. Its etiology spectrum spans a wide range, from benign lesions that do not require intervention (such as hormone use, endoscopic procedures, and chronic obstructive pulmonary disease) to critical lesions that require emergency surgery (such as mesenteric ischemia and intestinal necrosis), all of which can cause the same imaging manifestations.^1,2^ The biggest diagnostic challenge of PI lies in the highly similar imaging of benign and ischemic causes, while their treatment strategies are opposite. Delayed diagnosis can lead to extremely high mortality rates.^3,4^ The existing diagnostic system highly relies on the subjective experience of radiologists and lacks objective quantitative imaging grading standards, which poses a particularly prominent risk of missed diagnosis in emergency high-load scenarios. In addition, existing research lacks systematic integration of PI multimodal imaging performance, and artificial intelligence-assisted diagnosis is almost nonexistent in this field. Therefore, it is of great practical significance to systematically summarize the radiological imaging manifestations of PI and construct an AI-assisted auxiliary diagnostic system for improving diagnostic accuracy and guiding rapid clinical decision-making.

In terms of radiological imaging studies, existing research has accumulated a certain amount of information on the imaging features of PI, but the systematic and quantitative level is insufficient. In terms of x-ray plain films, Lassandro *et al.* analyzed the abdominal x-ray plain films of patients, which can detect intestinal wall gas accumulation, but the overall sensitivity of plain films is low, and a large number of early cases are missed.^5^ In terms of CT imaging, Graber *et al.* investigated the predictive factors for short-term mortality in patients with suspected mesenteric ischemia who underwent abdominal CT examination and showed PI, but this study did not include a systematic evaluation of accompanying signs.^6^ Fleck *et al.* included a total of 226 patients with intestinal wall enhancement (PI) detected by abdominal CT examination, and retrospectively extracted various variables during CT examination from the medical records. However, its research sample only comes from a single center, and its generalization is questionable.^7^ In summary, there are three core shortcomings in existing imaging research: the lack of systematic integration of multimodal imaging manifestations, the absence of accompanying signs in a unified evaluation framework, and the lack of an objective quantitative grading system validated by multiple centers.^8^

In the field of AI-assisted diagnosis research, deep learning technology has made significant progress in abdominal image analysis, but specific research on PI is still very scarce. In the detection of intestinal diseases, Spada *et al.* evaluated the non-inferiority of artificial intelligence (AI)-assisted image reading and standard image reading in the diagnosis of potential small bowel bleeding lesions (high P2 grade, medium P1 grade; Saurin grade).^9^ In the AI diagnosis of abdominal emergencies, Yao *et al.* explored the role of AI in enhancing the interpretation of acute abdominal images. They discussed how these technologies can assist in the detection and identification of acute abdominal diseases.^10^ Regarding image interpretability, Grad-CAM (Gradient-weighted Class Activation Mapping) and SHapley Additive exPlanations (SHAP) values have been introduced into abdominal CT diagnostic systems, enabling spatial visualization of model decisions and quantification of feature contributions. However, none of these studies directly addressed PI, and the few existing PI-related AI studies focus only on binary classification of single CT morphological features, without integrating accompanying signs or producing interpretable outputs, severely limiting their clinical applicability.^11^ In addition, existing models generally lack external multicenter validation, raising questions about their generalizability. The above research gaps indicate that building a PI-specific AI diagnostic system that integrates multidimensional imaging features, has interpretable outputs, has been rigorously validated, has urgent academic value, and addresses clinical needs.

In summary, based on the CT and x-ray imaging data of 612 multicenter PI patients, this study systematically summarized the multimodal imaging manifestations of PI and established a five-level PI-CT severity scoring system based on independent imaging predictive factors. On this basis, a two-level AI-assisted diagnostic system is constructed: The first level uses x-ray plain films for rapid screening, and the second level fuses gas morphology, accompanying signs, and clinical metadata from CT images, achieving accurate differentiation between benign and ischemic PI through an attention mechanism and providing interpretable output for model decision-making. The main innovation of this article is the first construction of a two-level framework that deeply integrates imaging pattern research and an AI diagnostic system for PI, providing reliable methodological support for rapid and accurate PI diagnosis in emergency scenarios. We hypothesized that a two-level AI system integrating x-ray triage, CT gas morphology, accompanying signs, and clinical metadata via cross-attention would improve discrimination between benign and ischemic PI compared with single-modality models, the PI-CT score alone, and radiologist assessments.

## II. RADIOLOGICAL MANIFESTATIONS OF PI (X-RAY PLAIN FILM AND CT)

Abdominal x-ray plain film is the most convenient imaging examination for the first visit in PI emergencies. Although its sensitivity is limited, identifying typical signs can provide important clues for clinical practice.^12,13^ On abdominal x-ray plain films, the direct signs of PI include a line-like translucent shadow (1.2 mm wide, corresponding to CT line-like gas, mostly in the submucosal layer) running along the intestinal wall, as well as a circular or oval cystic translucent shadow (215 mm in diameter, corresponding to CT cystic gas, mostly in the submucosal layer, commonly in benign PI) next to the intestinal wall. Those with a predominant small intestine distribution are more likely to indicate ischemic causes, while those with a predominant colon distribution are more common in benign causes. In the accompanying indirect signs, dendritic translucent shadows extending from the hepatic portal to the periphery of the liver indicate portal venous gas (PVG) are a strong predictor of intestinal ischemic necrosis.^14,15^ In addition, intestinal obstruction (stepped gas–liquid plane, intestinal dilation) or crescent-shaped free gas below the diaphragm (indicating intestinal perforation) can be seen.

To visually demonstrate the typical imaging features of PI in abdominal plain x-ray films and their corresponding relationship with CT signs, Fig. 1 was constructed in this paper. By comparing and analyzing features of different PI image types, this figure helps clarify the correlation between plain film signs and CT, providing an intuitive basis for subsequent PI imaging recognition and the development of AI-assisted diagnostic models.

**FIG. 1.**
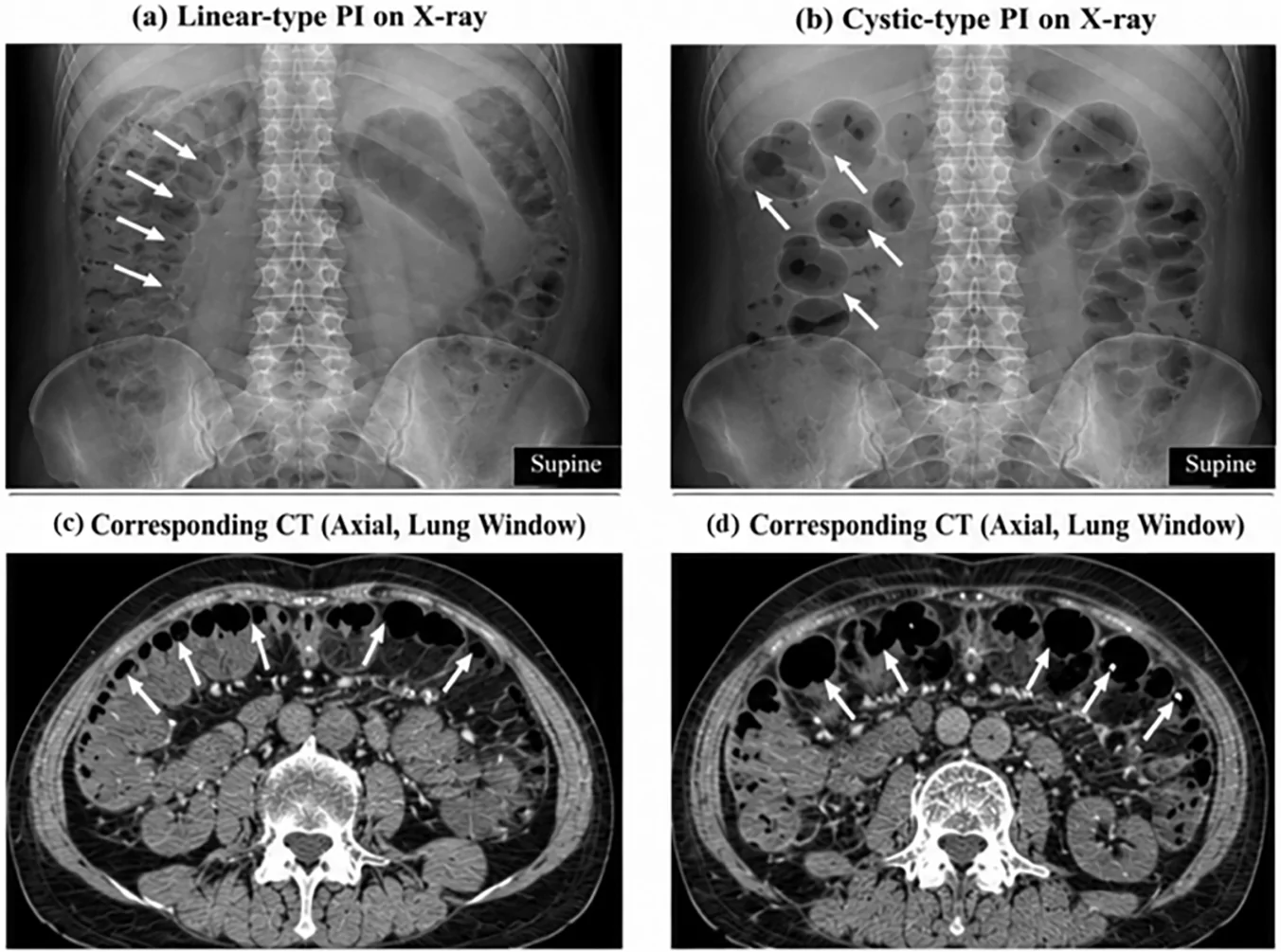
Typical x-ray manifestations of PI. (a) Linear PI on x-ray plain film; (b) cystic PI on x-ray plain film; (c) CT image corresponding to linear PI; and (d) CT images corresponding to cystic PI.

Figure 1 shows the characteristic features of two typical PI imaging types in abdominal x rays and corresponding CT scans. Figures 1(a) and 1(b) are abdominal x-ray plain films, showing linear PI and cystic PI, respectively. Figures 1(c) and 1(d) are CT axial images of the corresponding cases. Figures 1(a) and 1(c) show linear translucent shadows along the intestinal wall, corresponding to the submucosal linear gas accumulation in CT. Figures 1(b) and 1(d) show circular or oval vesicular gas accumulation shadows, corresponding to the accumulation of subserosal cystic gas. The arrow marks the key lesion area, clearly reflecting the morphological correspondence between different PI types in x ray and CT imaging.

CT is the preferred imaging method for diagnosing PI, as it clearly displays gas morphology, density, distribution range, intestinal wall layers, and various accompanying signs, providing a key basis for distinguishing benign from ischemic PI.^16,17^ The core CT imaging features of ischemic PI include the following: Gas morphology is divided into linear type (submucosal layer, CT value ranging from −900 to −700 HU, often indicating ischemia), cystic type (subserosal layer, CT value ranging from −950 to −850 HU, often seen in benign cases), and mixed type (the risk of ischemia increases when accompanied by PVG or decreased intestinal wall enhancement); in terms of distribution, ischemic PI is mostly diffuse (>2/3 of the intestinal segment) and involves the jejunum and ileum, while benign PI is mostly localized (<1/3) and mainly involves the colon, segmentally (1/3–2/3 of the intestinal segment); among the key accompanying signs, peripheral PVG,^18,19^ lack of intestinal wall enhancement (CT value decrease is >20 HU in the portal venous phase), diffuse mesenteric edema, and moderate to large amounts of ascites are all independent predictors of ischemic PI. In contrast-enhanced CT, the arterial phase reveals vascular embolism, the portal venous phase is best suited for assessing intestinal wall enhancement and PVG, and the delayed phase shows no enhancement, indicating the extent of intestinal necrosis. Together, these characteristics can be used to classify the etiology and severity of PI.

To systematically demonstrate the common and diagnostically valuable accompanying imaging features of PI on CT, this study selected four typical manifestations: PVG, mesenteric edema, abnormal intestinal wall enhancement, and ascites.^20,21^ These features can help determine the etiology and severity of PI and are closely related to adverse prognoses such as intestinal ischemia and necrosis. They form the basis for clinical-imaging evaluation and subsequent artificial intelligence model construction, as shown in Fig. 2.

**FIG. 2.**
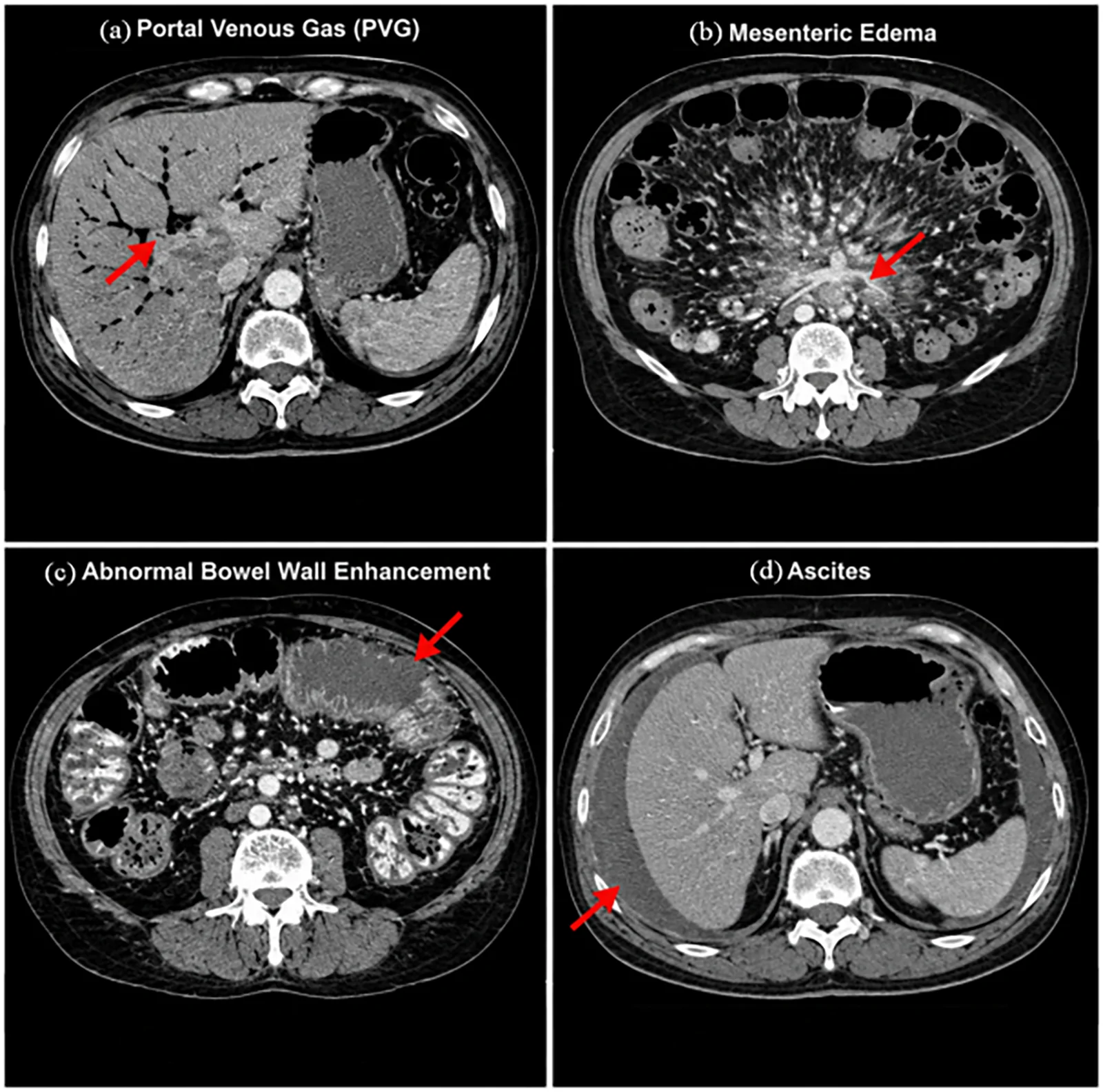
Typical manifestations of CT accompanying signs of PI. (a) PVG; (b) mesenteric edema; (c) abnormal enhancement of intestinal wall; and (d) ascites.

Figure 2 shows four typical accompanying signs in CT examination of PI patients. Figure 2(a) shows PVG, where the peripheral portal vein branches inside and outside the liver appear as dendritic low-density gas shadows; Fig. 2(b) shows mesenteric edema, characterized by increased density of mesenteric fat spaces, blurred vascular boundaries, and flocculent infiltration changes; Fig. 2(c) shows abnormal enhancement of the intestinal wall, indicating differences in the enhancement of the affected intestinal segment compared to the surrounding intestinal wall, suggesting abnormal blood supply status of the intestinal wall, which may be related to ischemic changes; Fig. 2(d) shows the presence of ascites, characterized by the accumulation of free low-density fluid in the abdominal cavity. The red arrows indicate the typical lesion areas of each imaging sign. These accompanying signs can reflect the degree of intestinal wall damage and mesenteric circulation status of PI patients from different perspectives, and have important clinical value for distinguishing benign PI from ischemic PI.

## III. CONSTRUCTION OF AI-ASSISTED DIAGNOSTIC SYSTEM

### A. Overall system design

This system adopts a two-level architecture: The first level is the x-ray plain film lightweight screening model, with the goal of maximizing sensitivity and determining the presence of PI signs in seconds, used for rapid triage and guiding priority CT examination. The second level is the CT multi-feature fusion precision diagnosis model, which combines three parallel branches (gas morphology, accompanying signs, and clinical metadata) with a cross-attention mechanism to output ischemic PI probability, PI-CT severity score, interpretable heatmap, and feature contribution, achieving diagnosis-level auxiliary decision-making.

In order to improve the accuracy of automated diagnosis of PI and fully integrate imaging features and clinical information, this study constructed a two-stage artificial intelligence-assisted diagnosis framework, as shown in Fig. 3. This framework first utilizes x-ray imaging for rapid screening, and then combines CT imaging and clinical data for refined analysis. Through multimodal feature fusion, classification of PI as benign or ischemic and risk assessment are achieved, providing reliable support for clinical decision-making.

**FIG. 3.**
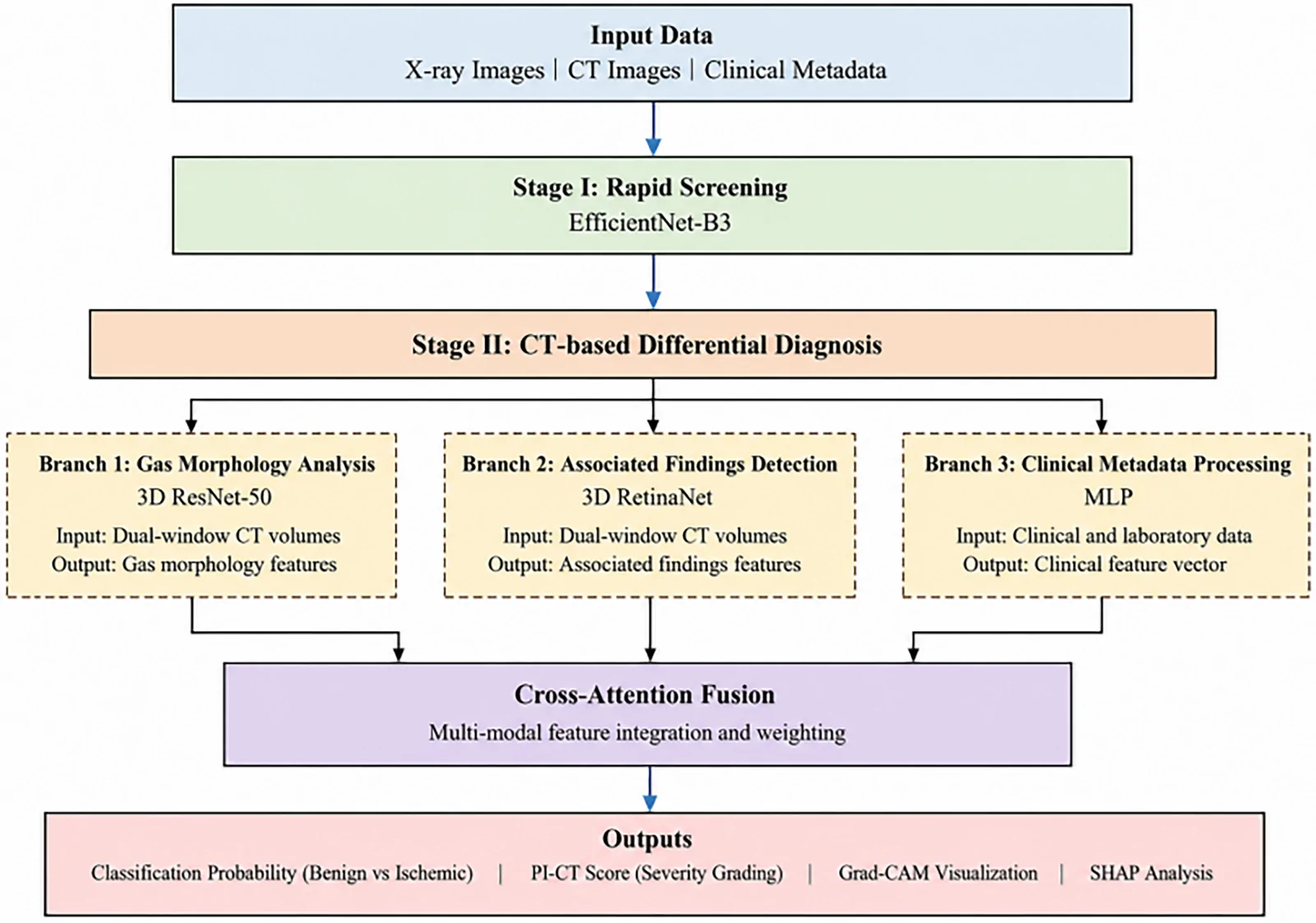
Overall architecture diagram of the two-level AI-assisted diagnostic system.

In Fig. 3, first, the input data include x-ray images, CT images, and clinical metadata. The first stage uses EfficientNet-B3 for rapid screening of x-ray images. For screened cases, the second stage is based on CT imaging for differential diagnosis, and three parallel analysis branches are set up: The gas speciation analysis branch utilizes 3D ResNet-50 to extract the spatial distribution characteristics of intestinal wall gas; the accompanying sign detection branch uses 3D RetinaNet to identify important imaging manifestations such as PVG, mesenteric edema, and ascites; and the clinical metadata branch uses a multilayer perceptron (MLP) to process laboratory test results and clinical indicators. Subsequently, the features extracted by each branch are fused and weighted through a cross-attention fusion module, ultimately outputting the probability of benign or ischemic PI classification, PI-CT severity score, and Grad-CAM and SHAP interpretability analysis results, achieving intelligent assisted diagnosis throughout the process, from image screening to risk assessment.

### B. First level: Rapid screening model for plain X-ray films

EfficientNet-B3 was used as the backbone for feature extraction.^22^ This network balances depth, width, and input resolution through a composite scaling method, and ImageNet-pretrained weights were used only for transfer initialization; the PI-sign classification task was trained and evaluated on our institutional x-ray dataset, not on ImageNet, with only about 12M parameters, making it very suitable as the backbone for rapid screening in the emergency department. Compared with larger models (such as ResNet-50 with 25M parameters), EfficientNet-B3 improves inference speed by nearly three times while maintaining high accuracy.

612 abdominal plain x-ray films (398 benign cases and 214 ischemic cases) corresponding to CT data were used. Each film was annotated by a senior radiologist with “whether there are typical PI signs on plain x-ray film.” Weights from the EfficientNet-B3 pre-trained dataset on ImageNet were loaded, with an initial learning rate of 3 × 10^−4^. For the first five epochs, the first two stages of the backbone network (Mobile Inverted Bottleneck Convolution blocks, MBConv blocks) were frozen, and only the classification head and high-level features were trained. Afterward, all layers were unfrozen for joint optimization.

Data Augmentation: Considering the large differences in x-ray image quality and varying exposure conditions, the following online augmentation strategy was adopted:

(1) Random brightness/contrast adjustment: A brightness adjustment factor in the range [0.8, 1.2] and a contrast adjustment factor in the range [0.7, 1.3] were used to simulate different x-ray exposure parameters.
(2) Simulating different exposure conditions: Gaussian noise (σ ≤ 0.05) and slight blur (Gaussian kernel size 1–3) were randomly added to enhance the model's robustness to image noise.
(3) Geometric transformations: Random horizontal flipping, random rotation ±10°, and random translation ±5% were used to simulate patient positional differences.

Preserving original details: Excessive cropping or scaling was avoided to prevent the loss of microbubbles.

Loss function: Weighted binary cross-entropy (WBCE) loss was used as the loss function,

$$L_{\text{WBCE}}=-\frac{1}{N}\sum_{i=1}^{N}\left[wy_{i}\text{ log }\left(p_{i}\right)+\left(1-y_{i}\right)\text{ log }\left(1-p_{i}\right)\right],\;w=2.0.$$ (1)

In formula (1), the weight w for positive samples (PI signs visible on x rays) is set to 2.0 to alleviate class imbalance due to their lower sample proportion in the dataset and improve the model's sensitivity to these cases.

Optimizer and scheduling: The Adam optimizer ( $\beta_{1}=0.9,\beta_{2}=0.999$) was used with an initial learning rate of 3 × 10^−4^. A StepLR (Step Learning Rate) scheduler was used to multiply the learning rate by 0.1 every 10 epochs. The total training epochs are 100, and the early stopping strategy (patience = 15) is based on the validation set AUC.

Inference time per image: <1 s/image on an NVIDIA A100 80GB GPU (Graphics Processing Unit) (approximately 0.8 s in actual testing). This speed includes image reading and preprocessing (0.1 s), model forward inference (0.6 s), and Grad-CAM heatmap generation (0.1 s). Even when deployed on lower-configuration GPUs (such as T4) or CPUs (such as Intel Xeon Gold 6248), the inference time can be controlled within 2–3 s, fully meeting the needs of real-time screening in emergency departments.

Clinical application scenarios: The model can be used as a plugin for PACS (Picture Archiving and Communication System), automatically triggering analysis for each abdominal x-ray film. When the probability is high (e.g., ≥0.7), it indicates the presence of typical PI signs on the x-ray film, prompting radiologists to prioritize the review and recommend further CT confirmation. It must be clearly stated that a low probability of x-ray signs (e.g., <0.3) does not rule out PI, as the overall sensitivity of x-ray films for PI is only about 30%–40%. Whether to perform a CT scan should be based on comprehensive clinical judgment and should not be determined independently by the model's threshold. This model is only an auxiliary prompting tool.

### C. Level two: CT multi-feature fusion precision diagnosis model

#### 1. CT image preprocessing

To ensure the comparability of multicenter, multi-device CT data and the stability of model training, the following standardized preprocessing procedure was performed on the original DICOM (Digital Imaging and Communications in Medicine) images:^23^

Dual window standardization (lung window/abdominal window in parallel): According to the evaluation framework in Sec. II, two standardized window images were generated simultaneously: The lung window had a window width of 1600 HU and a window level −600 HU. This window level highlights gas (CT value ranging from approximately −900 to −700 HU) for intestinal wall gas segmentation and morphological analysis. The abdominal window had a window width of 400 HU and a window level of 40 HU. This window level optimizes the display of soft tissue contrast for assessing accompanying signs such as intestinal wall enhancement, mesenteric edema, and ascites. Images from the two window levels are independently stored as single-channel 3D arrays and fed into different subsequent processing branches.

Resampling to a uniform voxel spacing: All CT sequences are resampled using trilinear interpolation to an anisotropic voxel spacing of 1 × 1 × 2 mm^3^. A 2 mm slice-thickness spacing is chosen to control computational load while maintaining sufficient Z-axis resolution (1 mm for fully isotropic images would result in a significant increase in data volume).

Intensity normalization: Z-score normalization is performed on images from each window level, in which the mean and standard deviation of the entire dataset are calculated, and each voxel value is converted to (x−μ)/σ. Normalization parameters are calculated independently for the lung and abdominal windows.

Data augmentation: Online augmentation strategies include random flipping—independent flipping along the X-, Y-, and Z-axes. Random rotation involves ±15° rotation around the Z-axis (cross section) and uses trilinear interpolation to fill the boundaries. Elastic deformation uses a grid-point random displacement field with a standard deviation of 0.2 to control the deformation intensity and simulate non-rigid deformation caused by respiratory movement and posture changes. Intensity perturbation—Gaussian noise (σ ≤ 0.05) and random contrast scaling (0.9–1.1 times) can be added to enhance the robustness of the model to differences in scanning parameters.

In order to improve the consistency and robustness of CT image input data, and fully preserve the key image information of intestinal wall gas accumulation and its accompanying signs, this study designed a standardized preprocessing process, as shown in Fig. 4. This process involves steps such as dual window level conversion, spatial resampling, intensity normalization, and data augmentation to unify the original DICOM data, thereby reducing the impact of different devices, scanning parameters, and individual patient differences, and providing high-quality input data for subsequent deep learning model training.

**FIG. 4.**
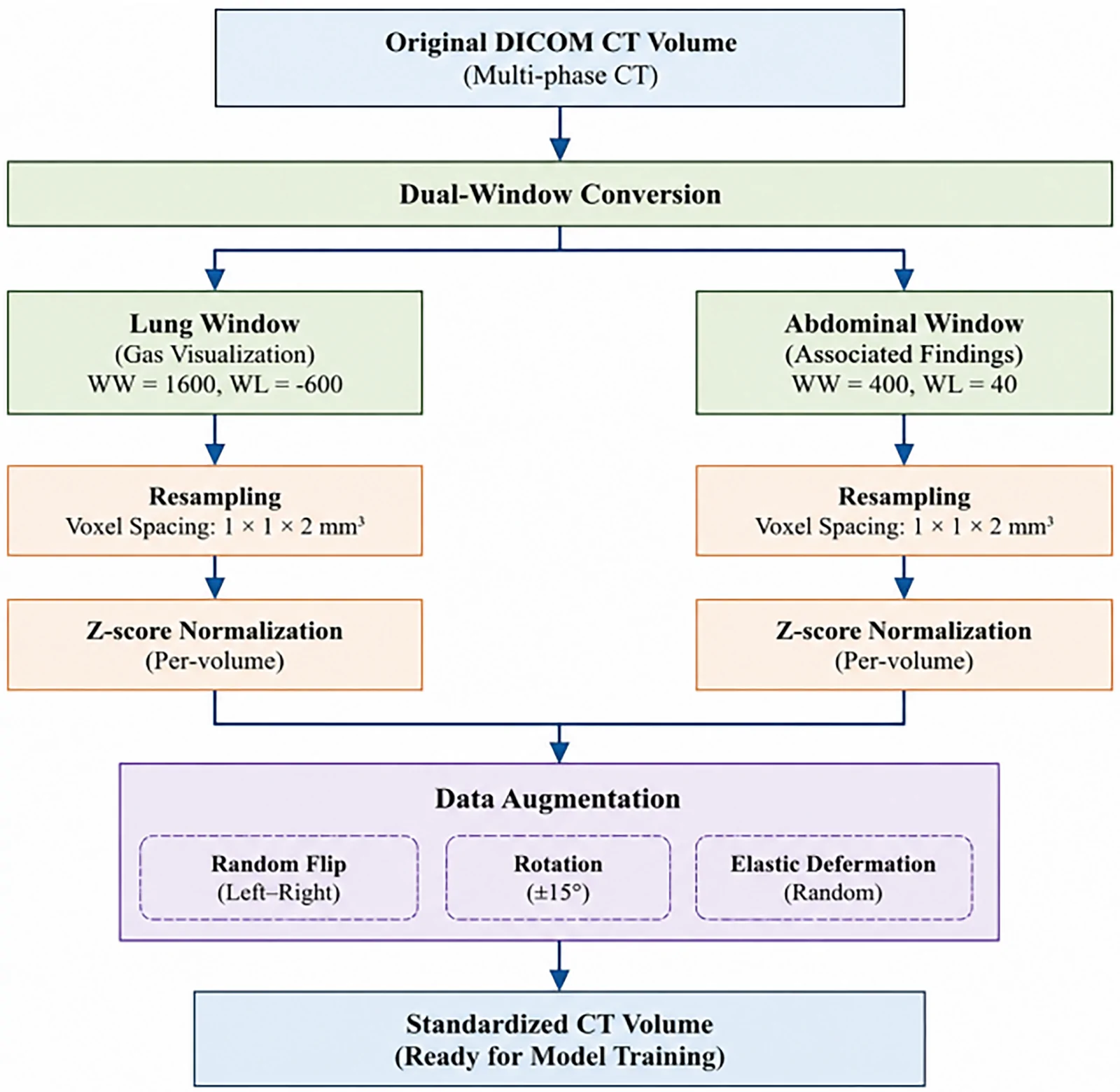
CT dual window level preprocessing and data enhancement flow chart.

In Fig. 4, first, the original DICOM CT data are input into the system and subjected to dual window level conversion to generate lung window images and abdominal window images, respectively, for highlighting intestinal wall gas accumulation and related accompanying signs. Subsequently, the two window level images were resampled separately to a uniform voxel spacing of 1 × 1 × 2 mm^3^, and Z-score normalization was used to standardize the grayscale distribution. After standardization, the dual window information is fused, and data augmentation operations such as Random Flip, Rotation (±15°), and Elastic Deformation are applied to improve the model's adaptability to anatomical changes and differences in scanning conditions. The final standardized CT volume serves as a unified input for subsequent segmentation, feature extraction, and classification model training.

#### 2. Intestinal wall gas automatic segmentation submodule (3D nnU-Net)

Accurate three-dimensional segmentation of intestinal wall gas underpins subsequent gas morphology feature extraction and quantitative analysis. Given the small volume (often <1 cm^3^), diverse shapes, and blurred boundaries between intestinal gas and adjacent tissues, this module adopts the 3D nnU-Net architecture,^24,25^ which performs well in medical image segmentation tasks and adapts to dataset characteristics.

Encoder: Adopting 5-level downsampling, each level contains 2 residual convolution blocks (ResBlocks); each residual block is composed of convolution+instance normalization+LeakyReLU (Leaky Rectified Linear Unit). The number of channels increases step by step: 32, 64, 128, 256, and 320. Downsampling uses convolution with a stride of 2 (kernel size 3 × 3 × 3), and the feature map spatial size is gradually halved. The encoder outputs a feature map size of 1/32 of the input patch (for a 128 × 128 × 64 input, output 4 × 4 × 2 × 320).

Decoder: The symmetric upsampling path uses transpose convolutions (upsampling factor 2 × 2 × 2) at each level to restore spatial resolution, and concatenates them with the corresponding encoder layer's feature maps through skip connections to preserve detail. After upsampling, we add two residual convolution blocks. To accelerate convergence and alleviate gradient vanishing, deep supervision is introduced, in which auxiliary segmentation losses are added to the last three levels of the decoder (i.e., upsampled to input sizes 1/8, 1/4, and 1/2), with weights of 0.25, 0.5, and 1.0, respectively. The main loss (final output layer) has a weight of 1.0.

Input Patch: Due to the extremely low proportion of PI gas in the entire CT volume (typically <0.1%), and to avoid GPU memory overflow, a random pruning strategy is adopted. The input patch size is 128 × 128 × 64 voxels (corresponding to approximately 128 × 128 × 128 mm^3^ in physical space). Only the lung window channel is used as input because the contrast between the gas (with the CT value ranging from −900 to −700 HU) and soft tissue in the lung window is optimal, and the background noise is minimal.

Loss function: To address severe class imbalance (the number of positive voxels is much smaller than the number of negative voxels), a combination of Dice Loss and Focal Loss is used,

$$L_{\text{seg}}=0.5\times L_{\text{Dice}}+0.5\times L_{\text{Focal}}.$$ (2)

The Dice Loss is given by

$$L_{\text{Dice}}=1-\frac{2|P\cap G|}{|P|+|G|}.$$ (3)

In Eq. (3), P is the predicted mask, and G is the gold standard. This loss directly optimizes the segmentation overlap and is insensitive to imbalance.

The Focal Loss is given by

$$L_{\text{Focal}}=-\sum_{i}{\alpha_{\text{bal}}(1-p_{i})}^{\gamma }\text{ log}(p_{i}).$$ (4)

In Eq. (4), γ = 2 and αbal = 0.75. This loss reduces the loss contribution of easily classifiable background pixels, forcing the model to focus on difficult samples (gas boundaries and tiny bubbles).

The combined loss allows the model to maintain both high recall (Dice part) and accurate boundary segmentation (Focal part).

Output: Generates a binary segmentation mask of the same size as the input patch (1: intestinal wall gas, 0: background), where “background” includes voxels of all non-intestinal wall gases (intestinal wall tissue, intestinal fluid/feces, mesentery, abdominal wall, bones, etc.). For the full CT volume, a sliding window strategy (step size half the patch size) is used for prediction. Overlapping regions are fused using a weighted average, ultimately outputting a complete 3D segmentation mask for intestinal wall gas. Segmentation performance was evaluated using Dice, IoU, and Recall.

#### 3. Three-branch parallel feature extraction

Branch 1: Gas Morphology Features (CNN (Convolutional Neural Network) Branch): The segmentation mask is element-wise multiplied with the original CT lung window image, forcing the model to extract features only from real gas regions. The backbone network uses 3D ResNet-50 (trained from scratch), with an input size of 128 × 128 × 64. The first-layer max pooling is removed to preserve spatial details, and a 2048-dimensional feature vector $f_{\text{morp}h}$ is output after global average pooling. The network automatically learns high-level semantic features such as gas morphology type (linear/cystic/hybrid), volume, CT value distribution, and spatial location.

Branch 2: Accompanying Sign Features (Detection Branch)

The input consists of portal venous phase CT abdominal window image (window width 400 HU, window level 40 HU). A 3D RetinaNet single-stage detection network extracts multi-scale features through Feature Pyramid Networks (FPN) and simultaneously performs classification and bounding box regression, making it particularly suitable for small and irregular accompanying signs. Detection targets include four categories: PVG (peripheral/hilar type), mesenteric edema (diffuse/localized), ascites (sparse/moderate/massive), and abnormal intestinal wall enhancement (diminished/absent). Output consists of presence probability and 3D bounding box coordinates for each sign, concatenated and then reduced to a 128-dimensional feature vector $f_{\_\text{sign}}$ via a fully connected layer.

Branch 3: Clinical Metadata (MLP Branch)

The input is a 5-dimensional clinical metadata vector, specifically including age (years), blood lactate (mmol/L), white blood cell count (WBC, ×10^9^/L), C-reactive protein (CRP, mg/L, the natural logarithm is taken before input due to skewed distribution), and time from onset to consultation (hours). The network structure is an MLP,^26^ a two-layer fully connected network with ReLU (Rectified Linear Unit) activation and batch normalization (BatchNorm) in the middle to accelerate training. Dropout is not used because the clinical features have low dimensionality (5 dimensions) and high information density, resulting in a low risk of overfitting. The input features are all Z-score standardized (based on the mean and standard deviation of the training set) before entering the network. The output is a 128-dimensional feature vector $f_{\text{clinical}}$, representing the patient's clinical status.

#### 4. Cross-attention multimodal fusion

Input projection: First, the two image features are concatenated into $f_{\text{img}}=[f_{\text{morp}h},\;f_{\text{sign}}]\in R^{2176}$, and then the dimension is reduced to 512 through linear projection to align with the head dimension of multi-head attention,

$$Q=W_{Q}f_{\text{clinical}}\;(Q\in R^{512}),\;K=W_{K}f_{\text{img }}(K\in R^{512}),\;V=W_{V}f_{\text{img}}\;(V\in R^{512}).$$ (5)

In formula (5), $W_{Q}$, $W_{K}$, and $W_{V}$ are learnable weight matrices.

Multi-Head Attention: Eight attention heads are set up, each with a dimension $d_{k}$= 512/8 = 64. Attention weights are calculated independently for each head,

$${\text{head}}_{i}=\text{Attention}(QW_{i}^{Q},\;KW_{i}^{K},\;VW_{i}^{V})=\text{softmax}(\frac{(QW_{i}^{Q}){\left(KW_{i}^{K}\right)}^{T}}{\sqrt{d_{k}}})(VW_{i}^{V}).$$ (6)

The outputs of 8 heads are concatenated and a linear transformation is performed to obtain the fused features,

$$f_{\text{fusion}}=\text{Concat}({\text{head}}_{1},\ldots ,\;{\text{head}}_{8})W^{O}\;(f_{\text{fusion}}\in R^{512}).$$ (7)

#### 5. Classification output header

Classifier structure: Fused feature diffusion (512 dimensions) is first reduced to 256 dimensions through a fully connected layer, followed by Dropout to prevent overfitting, and then mapped to 2 dimensions (benign/ischemic) through a second fully connected layer. Finally, the probabilities of the two categories are output through the Softmax function given by

$$y=\text{Sofemax}(\text{FC}(\text{Dropout}(\text{FC}(f_{\text{fusion}})))).$$ (8)

### D. Interpretability module

To enhance clinical doctors' trust in AI system decision-making and assist in verifying whether model behavior conforms to known PI imaging diagnostic rules, this system integrates two types of interpretability tools: spatial visualization (Grad-CAM) and feature contribution analysis (SHAP values).

Grad-CAM generates a three-dimensional heatmap of the same size as the input image by backpropagating the gradients of the final convolutional layer, highlighting the anatomical regions that the model is most concerned about during decision-making.^27^

The SHAP value is based on the Shapley value in game theory, which quantifies the marginal contribution of each input feature to the final prediction probability of the model.^28,29^ This system uses the TreeSHAP algorithm (applicable to tree models, but can use approximate kernel methods or DeepSHAP for neural networks) to interpret the fused classification results. SHAP values describe feature contributions but do not establish causality.

### E. Training configuration and optimization strategy

Both models are based on the PyTorch 2.0 framework and trained on dual NVIDIA A100 80GB GPUs. Due to the significant differences in task nature, data size, and network complexity between the first level (x-ray rapid screening model) and the second level (CT multi-feature fusion precision diagnosis model), they adopt different training configurations.

Level 1 (x-ray screening model): The optimizer uses Adam, the initial learning rate is set to 3 × 10^−4^, and the StepLR learning rate scheduling strategy is adopted. The learning rate is multiplied by 0.1 every 10 training epochs. The batch size can be set to 16, and the total number of training epochs is 100. The patience value of the early stopping strategy is 15, which means that when the AUC of the validation set does not improve for 15 consecutive epochs, the training is terminated and restored to the optimal weights. The model takes two-dimensional x-ray images as input, with few parameters and relatively lightweight training.

Second level (CT precision diagnosis model): Due to the large memory consumption and data volume of the 3D convolutional network, the optimizer uses AdamW (weight decay 1 × 10^−4^), with an initial learning rate of 1 × 10^−4^. The learning rate scheduling adopts cosine annealing with a period of 200 epochs, reducing the minimum learning rate to 1 × 10^−6^.

Inference speed: In deployment environments (such as a single NVIDIA A100 GPU), the first-level x-ray screening model can achieve millisecond-level inference, meeting the real-time screening needs of emergency departments. The complete process of the second-level CT precision diagnosis model takes slightly longer, mainly affected by the computational workload of the 3D segmentation and detection module. However, it remains acceptable in the clinical workflow and does not significantly delay report issuance. The specific inference performance can be systematically evaluated in subsequent prospective validation.

## IV. DATASET AND EXPERIMENTAL SETUP

### A. Data collection and patient admission and exclusion standards

This study is a multicenter retrospective study that collected consecutive cases of PI diagnosed clinically or pathologically from one tertiary hospital in North China, East China, and South China (referred to as Center A, Center B, and Center C, respectively) between January 2022 and June 2025. Due to the retrospective nature of the study and the de-identification of all data prior to analysis, patient informed consent was exempted.

Patients must meet the following conditions to be included in this study: PI confirmed by clinical findings (typical imaging findings+follow-up outcomes) or surgical pathology; complete abdominal x-ray plain film data (upright and supine positions) and abdominal enhanced CT data (including plain scan, arterial phase, portal phase); qualified CT image quality, with no significant motion or metal artifacts that affect the interpretation. Table I summarizes scanner/protocol differences and potential distribution shifts between development and external cohorts.

**TABLE I.** Dataset division and sample distribution details of each subset. In the CT scanner distribution, GE, Siemens, and Philips correspond to equipment from three internal centers. The external validation set uses equipment from a fourth hospital (United Imaging uCT 960+).

Dataset	Total N	Benign	Ischemic	Age (years)	CT scanner distribution (GE (General electric)/Siemens/Philips/Others)	Slice thickness (mm)	Center proportion
Training set	428	278	150	61.2 ± 13.8	142/143/143/0	1.25 ± 0.12	Center A: 35%, Center B: 33%, Center C: 32%
Validation set	92	60	32	61.5 ± 14.1	30/31/31/0	1.26 ± 0.11	Center A: 34%, Center B: 33%, Center C: 33%
Test set	92	60	32	60.9 ± 13.5	30/31/31/0	1.24 ± 0.13	Center A: 35%, Center B: 32%, Center C: 33%
External validation set	106	69	37	62.4 ± 14.2	0/0/0/106	1.27 ± 0.15	Center D: 100%

Those who meet any of the following conditions were excluded: severe artifacts in CT or x-ray images that affect the evaluation of intestinal wall gas or accompanying signs; primary intestinal tumors (such as colorectal cancer and lymphoma) that may interfere with imaging interpretation or etiological determination; more than 20% clinical data missing (including missing key indicators such as blood lactate and WBC); a history of major abdominal surgeries (such as gastrectomy, intestinal resection, or liver transplantation) that has led to changes in normal anatomical structures, affecting imaging evaluation.

According to the final clinical diagnosis and pathological results, all included patients were divided into two groups: (1) The first is a benign group (398 cases) meeting any of the following criteria: follow-up for more than 3 months, no surgical intervention, and spontaneous resolution or stabilization of PI signs; clearly caused by a benign etiology—such as history of hormone use (e.g., glucocorticoid therapy), post-endoscopic procedures (e.g., colonoscopy or polypectomy), or PI related to chronic obstructive pulmonary disease (COPD).^30,31^ No clinical or imaging evidence suggested intestinal ischemia or necrosis. (2) The ischemic group (214 cases) met at least one of the following criteria: direct intraoperative confirmation of intestinal necrosis (blackening of the intestinal wall, perforation, or pathological confirmation of necrotic tissue); digital subtraction angiography (DSA) confirming mesenteric vascular occlusion (superior mesenteric artery or vein thrombosis/embolism); or postoperative pathology indicating transmural infarction.

### B. Image annotation standards and quality control

To ensure gold-standard model training, this study annotated all CT and x-ray images at the pixel or region level and established a three-level review process.

Annotation tool: CT images are segmented at the 3D pixel level using 3D Slicer (version 5.2) to annotate the intestinal wall gas area and PVG area. The x-ray plain film is labeled with LabelImg (version 1.8) for bounding box annotation, outlining the lesion area (linear or cystic translucent shadow) for Grad-CAM supervision of the first-level screening model.

Annotation process: Primary annotation: Two resident physicians (with over 2 years of experience in radiology) who received unified training independently completed the preliminary annotation. CT outlines the gas boundary of the intestinal wall layer by layer, and suspicious lesions are selected on x rays. Review and correction: One attending physician (with over 5 years of experience in abdominal imaging) reviewed the primary annotation results case by case, correcting errors or missing areas. Final review confirmation: One associate chief physician (with over 10 years of experience in abdominal imaging) conducted a final review of the revised annotations, confirmed their accuracy, and include them in the dataset. Consistency sampling: In this study, 100 cases were randomly selected from all cases and independently relabeled by three senior doctors (2 associate chief physicians and 1 chief physician) to calculate the Kappa coefficient between each pair.

Label category (CT): Intestinal wall gas area (label 1) includes gas accumulation in the submucosal or subserosal layer, excluding free gas in the intestinal lumen. PVG area (label 2): Gas shadows in the intrahepatic portal vein branches, which need to be distinguished from biliary gas accumulation. Normal background (label 0): All other voxels.

Quality control: Weekly annotation meetings were held to collectively discuss and reach consensus on controversial cases. During the labeling process, 10 labeled cases were randomly inserted as quality control. If the consistency was lower than 0.80, they could be returned for relabeling.

### C. Data partitioning strategy

To objectively evaluate model performance and test its generalization ability, this study divided the dataset into an internal training/validation/testing set and an independent external validation set.

Internal dataset (612 cases): This article randomly divides the dataset into training, validation, and testing sets in a ratio of 7:1.5:1.5. Specifically, the training set consists of 428 cases (278 benign and 150 ischemic); validation set—92 cases (60 benign and 32 ischemic); and test set—92 cases (60 benign and 32 ischemic). Stratified sampling ensures that the benign/ischemic proportions in each subset match the overall population (approximately 65%: 35%). It also ensures that the x-ray and CT images from the same patient belong to the same subset, avoiding data leakage. We developed the second-stage CT model using all 612 confirmed PI patients with complete contrast-enhanced CT, not exclusively x-ray-positive cases. The training/validation/test sets comprised 428/92/92 cases, with benign: ischemic distributions of 278:150, 60:32, and 60:32.

External validation set: A total of 106 PI patients (69 benign and 37 ischemic) were independently collected from a fourth tertiary hospital (Central China region, January 2023 to June 2025) with the same inclusion and exclusion criteria. This external validation set is independent of the entire model development process and is only used for final generalization performance evaluation.

In order to comprehensively demonstrate the specific composition of the internal training, validation, testing set, and external validation set, ensure the independence of model training, parameter tuning, internal evaluation, and generalization ability verification, and clarify the distribution balance of each subset in terms of demographic characteristics, imaging devices, and source centers, this study summarizes the detailed division of the dataset in Table I.

In Table I, the internal dataset contains 612 cases, divided into a training set (428 cases), a validation set (92 cases), and a test set (92 cases) in a 7:1.5:1.5 ratio. The benign-to-ischemic ratio remained approximately 65%:35% across all subsets. The mean age was similar across subsets (60.9–61.5 years), and the CT scanner distribution (GE, Siemens, Philips) was balanced among the three internal centers. The slice thickness was controlled at approximately 1.25 mm. The external validation set included 106 independent cases from a fourth center (Central China, using United Imaging uCT 960+), comprising 69 benign cases and 37 ischemic cases, with a mean age of 62.4 years. This partitioning ensured the separation of model development from independent validation, and the data partitioning was reasonable, effectively supporting subsequent model training and generalization performance evaluation.

### D. Baseline model setup

To objectively evaluate the performance advantages of the proposed two-level fusion model in the differential diagnosis of benign and ischemic PI, five representative baseline models were set up, covering different technical approaches such as single-modality, traditional machine learning, pure Transformer architecture, and pure image rule scoring. All baseline models were trained or parameterized on the same training set (428 cases), and their performance was evaluated on the same internal test set (92 cases) and external validation set (106 cases).

Baseline 1 (Single Morphological Feature Model): This model only uses gas morphological information from CT lung window images and does not include any accompanying signs or clinical metadata. The backbone network adopts 3D ResNet-50, with the input being a gas region guided by a segmentation mask (the same as the second-level branch in this article), and the output being a binary classification probability. This baseline aims to validate the diagnostic upper limit that relies solely on gas morphology characteristics.

Baseline 2 (morphology+accompanying sign model): Based on baseline 1, accompanying sign features are added. Specifically, the feature vectors output from the gas morphology branch (3D ResNet-50) and the accompanying sign detection branch (3D RetinaNet) are directly concatenated and fed into the classification head (fully connected layer + Softmax), but no clinical metadata is included. This baseline evaluates the gain from multi-feature fusion (morphology + accompanying signs) within the image and verifies whether clinical metadata provides irreplaceable information.

Baseline 3 [traditional radiomics + SVM (support vector machine) model]: The traditional radiomics workflow is adopted: First, radiologists manually delineate regions of interest such as intestinal wall gas and PVG, and use PyRadiomics to extract 107 three-dimensional features including morphology, first-order statistics, and texture; then, feature selection is performed using LASSO (Least Absolute Shrinkage and Selection Operator) regression; finally, a linear support vector machine (SVM) is used for classification.^32,33^ This baseline is used to compare the performance differences between deep learning end-to-end methods and manual feature + traditional classifier.

Baseline 4 (Vision Transformer Base 16 × 16, ViT-B/16 model): A pure Transformer architecture, the 3D Vision Transformer, is used as the classifier. The input is a cropped block of a CT lung window image (the gas region is expanded to a fixed size of 64 × 64 × 32 voxels), with no convolutional inductive bias. This baseline verifies whether CNN architectures (such as ResNet-3D) still outperform Transformers in this task, or whether each has its own advantages and disadvantages.

Baseline 5 (PI-CT scoring system only): This baseline uses no deep learning model and relies entirely on the five-level PI-CT severity scoring system established in Sec. V (Table IV). A score (0–12 points) is automatically calculated for each patient according to CT image rules. We use this baseline to evaluate the diagnostic efficacy gap between pure image rule scoring and the AI model in this paper, verifying whether the AI can surpass the interpretative ability of fixed rules.

Baseline 6 (published clinical-radiological score): We refit/revalidate a previously published PI clinical-imaging risk score on the same training and test splits as an independent external baseline.

The model presented in this article (two-level fusion complete system) is the two-level AI-assisted diagnostic system described in Sec. III. The first level is the x-ray plain film rapid screening model based on EfficientNet-B3, and the second level is the three-branch cross-attention precision diagnosis model that integrates gas morphology, accompanying signs, and clinical metadata. This model simultaneously outputs ischemic PI probability and the PI-CT score, forming the complete method proposed in this paper.

### E. Experimental design for ablation

To evaluate the independent contributions of each key module in the two-level fusion model proposed in this article, six ablation experiments were designed. By removing or replacing specific components one by one based on the complete model, the impact of each module on the final diagnostic performance was quantitatively analyzed. All ablation configurations use the same training, validation, and testing sets as the complete model, and maintain consistent hyperparameters to ensure fairness in comparison.

Remove x-ray screening level (only CT precision diagnosis): It can skip the first-level x-ray screening model and directly input CT images into the second-level precision diagnosis model. This configuration is used to verify the necessity of a two-level cascaded architecture and evaluate whether x-ray screening can provide rapid triage value without significant loss of accuracy.

Remove gas morphology branch: In the second-level precision diagnosis model, the gas morphology feature extraction branch (3D ResNet-50) can be removed, and only the accompanying sign branch and clinical metadata branch can be retained for classification after fusion. This configuration is used to verify the core role of gas morphology characteristics in differential diagnosis.

Remove the accompanying sign branch: The accompanying sign detection branch (3D RetinaNet) can be removed, leaving only the gas morphology branch and clinical metadata branch. This configuration is used to evaluate the enhancement of model performance by accompanying signs such as PVG and mesenteric edema.

Remove clinical metadata branch: The clinical metadata branch (MLP) can be removed, leaving only two imaging branches—gas morphology and accompanying signs. This configuration is used to verify whether clinical indicators such as age and blood lactate can provide independent predictive information beyond imaging features.

Remove cross-attention (changed to simple concatenation): All features of the three branches mentioned above can be retained, but the feature vectors output by the three branches are directly concatenated and sent to the classification head, replacing the cross-attention fusion mechanism proposed in this paper. This configuration is used to verify whether the attention mechanism can effectively learn the cross-modal interaction relationship of “dynamically weighted image features under clinical background guidance.”

Complete model (method described in this article): All modules, namely, the first-level x-ray screening + second-level three-branch cross-attention fusion precision diagnosis model, can be retained as a benchmark for comparison.

### F. Definition of evaluation indicators

To comprehensively evaluate the performance of the model, this study defines evaluation indicators from four dimensions: classification efficiency, probability calibration, clinical comparability, and statistical testing. All indicators were calculated on an internal test set (92 cases) and an external validation set (106 cases), and compared using corresponding statistical tests.

Classification performance metrics: AUC (Area Under the ROC (Receiver Operating Characteristic)) reflects the model's intrinsic ability to distinguish between positive and negative samples, and is not affected by thresholds.^34,35^ AUC values range from [0.5, 1], where 0.5 indicates no discriminatory ability and 1 indicates perfect classification.

Sensitivity (Recall): The true positive rate, i.e., the proportion of ischemic PI patients correctly identified as ischemic,

$$\text{Sensitivity}=\frac{\text{TP}}{\text{TP}+\text{FN}}.$$ (9)

Here, TP (True Positive) is the number of correctly predicted cases of ischemia, which is a true positive; FN (False Negative) refers to the number of ischemic cases that were incorrectly predicted as benign, resulting in false negatives or missed diagnoses.

Specificity: True negative rate, which refers to the proportion of benign PI patients correctly identified as benign,

$$\text{Specificity}=\frac{\text{TN}}{\text{TN}+\text{FP}}.$$ (10)

Here, TN (True Negative) is the number of cases correctly predicted as benign and is a true negative; FP (False Positive) is the number of benign cases that were incorrectly predicted as ischemic, which is a false positive/misdiagnosis.

PPV (Positive Predictive Value): The proportion of patients predicted by the model to be truly ischemic,

$$\text{PPV}=\frac{\text{TP}}{\text{TP}+\text{FP}}.$$ (11)

NPV (Negative Predictive Value): The proportion of patients predicted by the model to be truly benign,

$$\text{NPV}=\frac{\text{TN}}{\text{TN}+\text{FN}}.$$ (12)

F1 score: The harmonic mean of sensitivity and PPV, which comprehensively evaluates the performance of the model,

$$F_{1}=2\times \frac{\text{Sensitivity}\times \text{PPV}}{\text{Sensitivity}+\text{PPV}}.$$ (13)

Calibration performance metric: Brier Score measures the mean squared error between the predicted probability and the true label (0 or 1),^36,37^

$$\text{Brier}=\frac{1}{N}\sum_{i=1}^{N}{\left(p_{i}-y_{i}\right)}^{2}.$$ (14)

Here, N is the total number of samples, pi is the probability of the model predicting the i-th sample as ischemic, and y_i_ is the true label (ischemic = 1, benign = 0). The value range of the Brier Score is [0,1], and the smaller the value, the more accurate the probability calibration (0 is perfect, 0.25 is a model without information).

Clinical comparative evaluation: To verify the diagnostic advantages of the proposed model compared with human radiologists, this paper compared the model's classification performance with that of doctors of different seniority. Diagnostic efficacy of doctors: This article invites three radiologists (junior: 2 years of experience; intermediate: 5 years of experience; senior: 10 years of experience) to independently review CT images of 92 patients in the test set, and each doctor provides a binary judgment of benign/ischemic. We can calculate and compare the AUC, sensitivity, specificity, PPV, NPV, F1 score, and Brier score for each doctor with the model presented in this article.

Statistical testing method: DeLong's test^38^ is used to compare whether there is a statistically significant difference in the ROC and AUC between two related ROC tests. This test calculates the Z-statistic and its corresponding P-value by estimating the covariance matrix of AUC. This study used the DeLong test to pairwise compare the AUC differences between the proposed model and various baseline models, as well as among doctors of different seniority. The significance level is set to α = 0.05 (bilateral), and P < 0.05 is considered statistically significant.

## V. RESULT ANALYSIS

### A. Baseline clinical characteristics of patients

In order to clarify the baseline differences in demographic characteristics, underlying diseases, laboratory indicators, and clinical outcomes between benign and ischemic PI patients, and to provide clinical background references for subsequent imaging feature analysis and AI model construction, this study compared key variables between the two groups of patients, and the results are summarized in Table II.

**TABLE II.** Comparison of baseline clinical characteristics among 612 patients. Independent sample t-test (statistic t) is used for continuous variables that conform to a normal distribution, and Mann–Whitney U test (statistic Z) is used for skewed distributions. The categorical variables are tested using the chi-square test (chi-square statistic). CRP is represented by the median (interquartile range).

Category	Variable	Benign group (n = 398)	Ischemic group (n = 214)	Statistic (χ^2^/t/Z)
Demographic characteristics	Age (years)	58.3 ± 14.6	65.7 ± 12.9	t = 6.21
	Sex (male/female)	208/190	121/93	χ^2^ = 1.38
	BMI (Body mass index) (kg/m^2^)	23.1 ± 3.2	22.4 ± 3.0	t = 2.67
Comorbidities	Diabetes mellitus, n (%)	86 (21.6%)	79 (36.9%)	χ^2^ = 16.24
	Chronic obstructive pulmonary disease, n (%)	54 (13.6%)	18 (8.4%)	χ^2^ = 3.65
	History of corticosteroid use, n (%)	47 (11.8%)	9 (4.2%)	χ^2^ = 9.82
	Hypertension, n (%)	112 (28.1%)	84 (39.3%)	χ^2^ = 7.88
Laboratory parameters	Blood lactate (mmol/L)	1.7 ± 0.8	4.2 ± 1.9	Z = 11.43
	White blood cell count (×10^9^/L)	9.2 ± 3.5	14.8 ± 5.6	Z = 9.87
	C-reactive protein (mg/l)	18.6 (6.3–42.1)	87.4 (34.2–156.3)	Z = 10.21
Clinical outcomes	Surgery rate, n (%)	12 (3.0%)	187 (87.4%)	χ^2^ = 432.5
	Length of hospital stay (days), median (interquartile range, IQR)	6 (4–10)	14 (9–23)	Z = 11.05
	30-day mortality, n (%)	2 (0.5%)	38 (17.8%)	Χ^2^ = 68.34

In Table II, the average age of patients in the ischemic group is significantly higher than that in the benign group (65.7 years vs 58.3 years). They have a higher prevalence of diabetes (36.9% vs 21.6%) and hypertension (39.3% vs 28.1%), while the history of hormone use is more common in the benign group (11.8% vs 4.2%). In terms of laboratory indicators, the levels of blood lactate, WBC, and C-reactive protein were significantly increased in the ischemic group. In terms of clinical outcomes, the surgical rate (87.4% vs 3.0%), hospital stay (median 14 days vs 6 days), and 30-day mortality rate (17.8% vs 0.5%) in the ischemic group were significantly higher than those in the benign group. The above results suggest that ischemic PI patients have older age, more cardiovascular complications, more significant inflammation and lactate elevation, and significantly poorer clinical prognosis, providing a reliable clinical data basis for the establishment of multifactorial prediction models and PI-CT scoring systems in the future.

### B. PI-CT severity five-level scoring system

#### 1. Screening of independent critical predictive factors

To construct a PI-CT severity scoring system, independent image predictive factors related to ischemic PI were first screened. A univariate logistic regression analysis was conducted on 612 patients with PI, using CT imaging features (gas morphology, distribution range, PVG, abnormal intestinal wall enhancement, mesenteric edema, ascites) as independent variables and ischemic PI as the dependent variable. Univariate analysis showed that the following characteristics were significantly associated with ischemic PI (P < 0.05): linear gas (vs cystic), diffuse distribution (vs localized), PVG (peripheral vs absent), abnormal intestinal wall enhancement (absent vs normal), diffuse mesenteric edema (vs absent), and moderate to large ascites (vs absent).

These six univariately significant variables were included in a multivariate logistic regression model, and independent predictors were screened using a stepwise forward method. All six variables were retained in the model, and their regression coefficients (β), odds ratios (OR), and 95% confidence intervals (CI) are shown in Table III.

**TABLE III.** Independent predictors of ischemic PI identified by multivariate logistic regression analysis. This table uses binary variable coding. Each predictor only compares extreme outcomes with the control/no results. Intermediate types (such as mixed gas, segmental distribution, hilar PVG, decreased intestinal wall enhancement, localized mesenteric edema, and small amount of ascites) did not reach a significant level in univariate analysis (P > 0.05) and were therefore not included in the multivariate model. The reference groups for each variable are as follows: cystic gas, localized distribution, no PVG, normal intestinal wall enhancement, no mesenteric edema, and no ascites.

Independent predictor	β coefficient	OR	95% CI
Gas morphology (linear vs. cystic type)	1.90	6.7	3.9–11.5
Distribution (diffuse vs. localized)	1.63	5.1	2.8–9.3
Portal venous gas (peripheral type vs. none)	3.35	28.4	15.2–52.8
Abnormal bowel wall enhancement (absent vs. normal)	2.72	15.2	8.9–25.9
Mesenteric edema (diffuse vs. none)	2.12	8.3	4.6–15.0
Ascites (moderate to large vs. none)	1.10	3.0	1.7–5.3

In Table III, peripheral PVG was the strongest independent predictor of ischemic PI (OR = 28.4, 95% CI: 15.2–52.8), followed by absent intestinal wall enhancement (OR = 15.2, 95% CI: 8.9–25.9), diffuse mesenteric edema (OR = 8.3, 95% CI: 4.6–15.0), linear gas (OR = 6.7, 95% CI: 3.9–11.5), diffuse distribution (OR = 5.1, 95% CI: 2.8–9.3), and moderate to large ascites (OR = 3.0, 95% CI: 1.7–5.3). All six imaging features were significantly and independently associated with ischemic PI (the lower limit of the 95% confidence interval for all Ors was greater than 1), providing core variables for the subsequent construction of a PI-CT severity scoring system.

#### 2. Scoring system construction and weighting

To maintain the integrity of the scoring levels for each item (each with three levels: 0, 1, and 2), this study assigned a score of 0–2 (maximum 2 points) to each of the six independent predictors, with a total score range of 0–12 points. Detailed criteria and weightings for each scoring item are shown in Table IV. Patients were divided into five levels based on their total score, with each level corresponding to the following clinical management recommendations: Grade I (0–3 points), highly likely benign—for which follow-up observation is recommended; Grade II (4–5 points), low suspicion—close follow-up is recommended (e.g., repeat CT scan within 48 h); Grade III (6–7 points), moderate suspicion—aggressive treatment is recommended (hospitalization and completion of relevant examinations); Grade IV (8–9 points), highly suspicious—surgical consultation is recommended to assess surgical indications; Grade V (10–12 points) (highly suggestive of ischemic PI)—emergency surgical evaluation is recommended.

**TABLE IV.** Detailed standards for the five-level severity scoring system for PI-CT.

Scoring item	Criteria	Points
Gas morphology	0 points: cystic type	2
	1 point: mixed type	
	2 points: linear type	
Distribution	0 points: localized (<1/3 bowel segment)	2
	1 point: segmental (1/3–2/3 bowel segment)	
	2 points: diffuse (>2/3 bowel segment)	
Portal venous gas	0 points: none	2
	1 point: hilar type (limited to main portal vein or first-order branches)	
	2 points: peripheral type (extending to subcapsular second-order or more distal branches)	
Abnormal bowel wall enhancement	0 points: normal (target sign present)	2
	1 point: decreased enhancement (20%–50% reduction)	
	2 points: absent enhancement	
Mesenteric edema	0 points: none	2
	1 point: localized or segmental (<50% of mesentery involved)	
	2 points: diffuse (≥50% of mesentery involved)	
Ascites	0 points: none	2
	1 point: small (only in pouch of Douglas or hepatorenal recess)	
	2 points: moderate to large (scattered or entire peritoneal cavity)	
Total score	0–12 points	⋯

In Table IV, each predictive factor (gas morphology, distribution range, PVG, abnormal intestinal wall enhancement, mesenteric edema, ascites) was scored on a scale of 0, 1, and 2, with a total score range of 0–12 points. This scoring system accounts for intermediate-risk gradients and provides an intuitive, quantitative reference for clinical decision-making.

Based on the total score (0–12), patients are classified into five grades: Grade I (0–3), highly likely benign—follow-up observation is recommended; Grade II (4–5), low suspicion—close follow-up is recommended; Grade III (6–7), moderate suspicion—hospitalization and further evaluation are recommended; Grade IV (8–9), highly suspicious—surgical consultation is recommended; Grade V (10–12), highly suggestive of ischemic PI—emergency surgical evaluation is recommended.

### C. Statistical analysis results of radiographic imaging features

To further clarify differences in multimodal imaging features between benign and ischemic PI, this study systematically compared the x-ray plain films and CT signs of 612 patients and calculated the odds ratio (OR) and 95% confidence interval (CI) for each sign. The results are detailed in Table V.

**TABLE V.** Comparative analysis of multimodal imaging features between two groups of patients. The value of 1.0 (ref) indicates that this category is used as the reference group, and its odds ratio (OR) is set to 1.0.

Category	Feature	Benign group (n = 398)	Ischemic group (n = 214)	OR (95% CI)
**X-ray plain film**	Linear lucency, n (%)	53 (13.3)	112 (52.3)	7.1 (4.8–10.5)
	Cystic lucency, n (%)	86 (21.6)	31 (14.5)	0.6 (0.4–0.9)
	Portal venous gas on x-ray, n (%)	2 (0.5)	38 (17.8)	43.2 (10.3–181.5)
	X-ray positive rate, %	31.2	68.2	4.7 (3.3–6.7)
**CT features**: Gas morphology	Cystic type, n (%)	224 (56.3)	28 (13.1)	1.0 (ref)
	Mixed type, n (%)	50 (12.6)	29 (13.6)	1.1 (0.6–1.9)
	Linear type, n (%)	124 (31.2)	157 (73.4)	6.7 (3.9–11.5)
	Mean CT value (HU)	−892 ± 45	−876 ± 52	⋯
	Gas volume (cm^3^), median (IQR)	0.8 (0.3–2.1)	5.6 (2.4–12.7)	⋯
Involved bowel segment	Localized (<1/3), n (%)	268 (67.3)	42 (19.6)	1.0 (ref)
	Segmental (1/3–2/3), n (%)	98 (24.6)	68 (31.8)	1.7 (1.0–2.9)
	Diffuse (>2/3), n (%)	32 (8.0)	104 (48.6)	5.1 (2.8–9.3)
	Portal venous gas (CT), n (%)	13 (3.3)	125 (58.4)	28.4 (15.2–52.8)
Bowel wall enhancement	Normal, n (%)	356 (89.4)	21 (9.8)	1.0 (ref)
	Decreased, n (%)	28 (7.0)	38 (17.8)	2.7 (1.6–4.6)
	Absent, n (%)	14 (3.5)	155 (72.4)	15.2 (8.9–25.9)
	Mesenteric edema, n (%)	34 (8.5)	136 (63.6)	8.3 (4.6–15.0)
	Ascites, n (%)	68 (17.1)	138 (64.5)	3.0 (1.7–5.3)
	PI-CT score (0–12)	2.1 ± 1.1	7.2 ± 1.4	⋯
Overall sensitivity	Overall sensitivity of CT, %	⋯	⋯	96.2%
	Overall sensitivity of x ray, %	⋯	⋯	38.7%

Table V compares the differences in radiographic and CT imaging features between patients with benign and ischemic PI. In the ischemic group, the incidence of linear gas (73.4% vs 31.2%), diffuse distribution (48.6% vs 8.0%), PVG (58.4% vs 3.3%), absent intestinal wall enhancement (72.4% vs 3.5%), diffuse mesenteric edema (63.6% vs 8.5%), and moderate to large ascites (64.5% vs 17.1%) were significantly higher than in the benign group, with corresponding odds ratios (OR) of 6.7, 5.1, 28.4, 15.2, 8.3, and 3.0, respectively. Conversely, cystic gas was more common in the benign group (56.3% vs 13.1%). The overall diagnostic sensitivity of plain x ray for PI was only 38.7%, far lower than CT's 96.2%, further confirming that CT is the preferred imaging method for diagnosing PI.

### D. Results of the Level 1 X-ray screening model

To visually demonstrate the core performance indicators of the Level 1 plain x-ray rapid screening model (EfficientNet-B3) on the internal test set, including sensitivity, specificity, and inference speed, and to verify the feasibility and accuracy of this model in rapidly triaging PI in emergency scenarios, Fig. 5 clearly compares and assesses the model's balance between reducing the missed diagnosis rate (high sensitivity) and reducing unnecessary CT examinations (high specificity), while also clarifying whether its second-level inference speed meets real-time clinical needs.

**FIG. 5.**
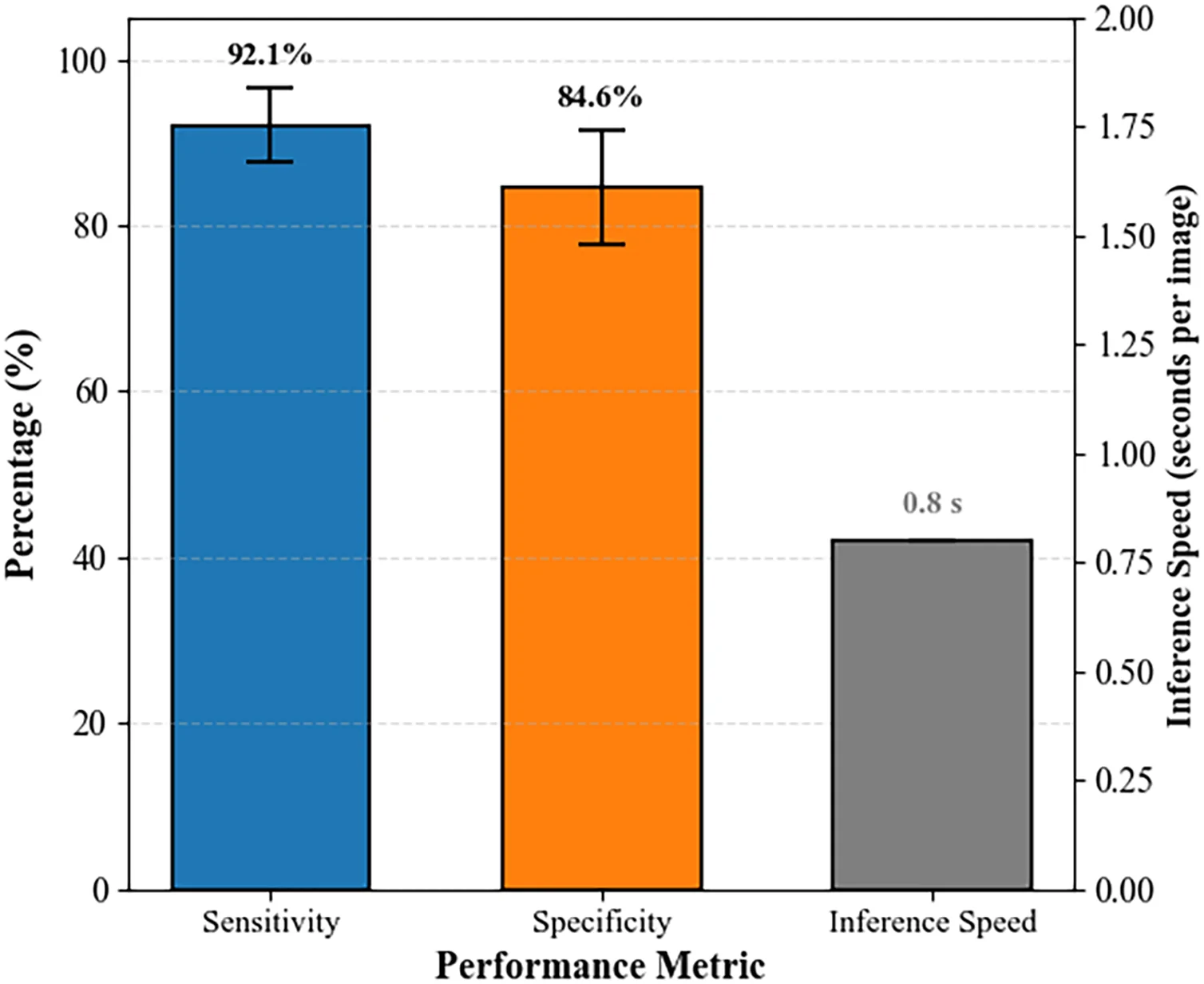
Performance bar chart of the first-level x-ray screening model.

In Fig. 5, the sensitivity is 92.1% (95% CI: 87.6%–96.6%), the specificity is 84.6% (95% CI: 77.8%–91.4%), and the inference speed is 0.8 s per sheet. The error line represents the 95% confidence interval. The results showed that the model achieved over 92% sensitivity while maintaining high specificity, and the inference time for a single image was under 1 s, meeting the requirements for rapid emergency screening.

The negative predictive value (NPV) of the Level-1 x-ray screening model was 93.2% in the internal test set. The negative class was confirmed PI patients without visible typical PI signs on x ray, not PI-free individuals. Therefore, the reported specificity (84.6%) reflects recognition of the absence of x-ray signs among confirmed PI patients and should not be interpreted as screening specificity in a general or clinically suspected population.

### E. Second-level CT precision diagnosis model

To comprehensively evaluate the diagnostic performance of the constructed CT precision diagnosis model in the differentiation of benign and ischemic PI, and objectively compare its differences with different baseline models and radiologists with different levels of experience, this study used receiver operating characteristic (ROC) curves for comprehensive analysis.^39,40^ The results are shown in Fig. 6. By drawing ROC curves of various models and doctors, the performance advantages and clinical application potential of artificial intelligence models in PI ischemia risk identification can be visually demonstrated.

**FIG. 6.**
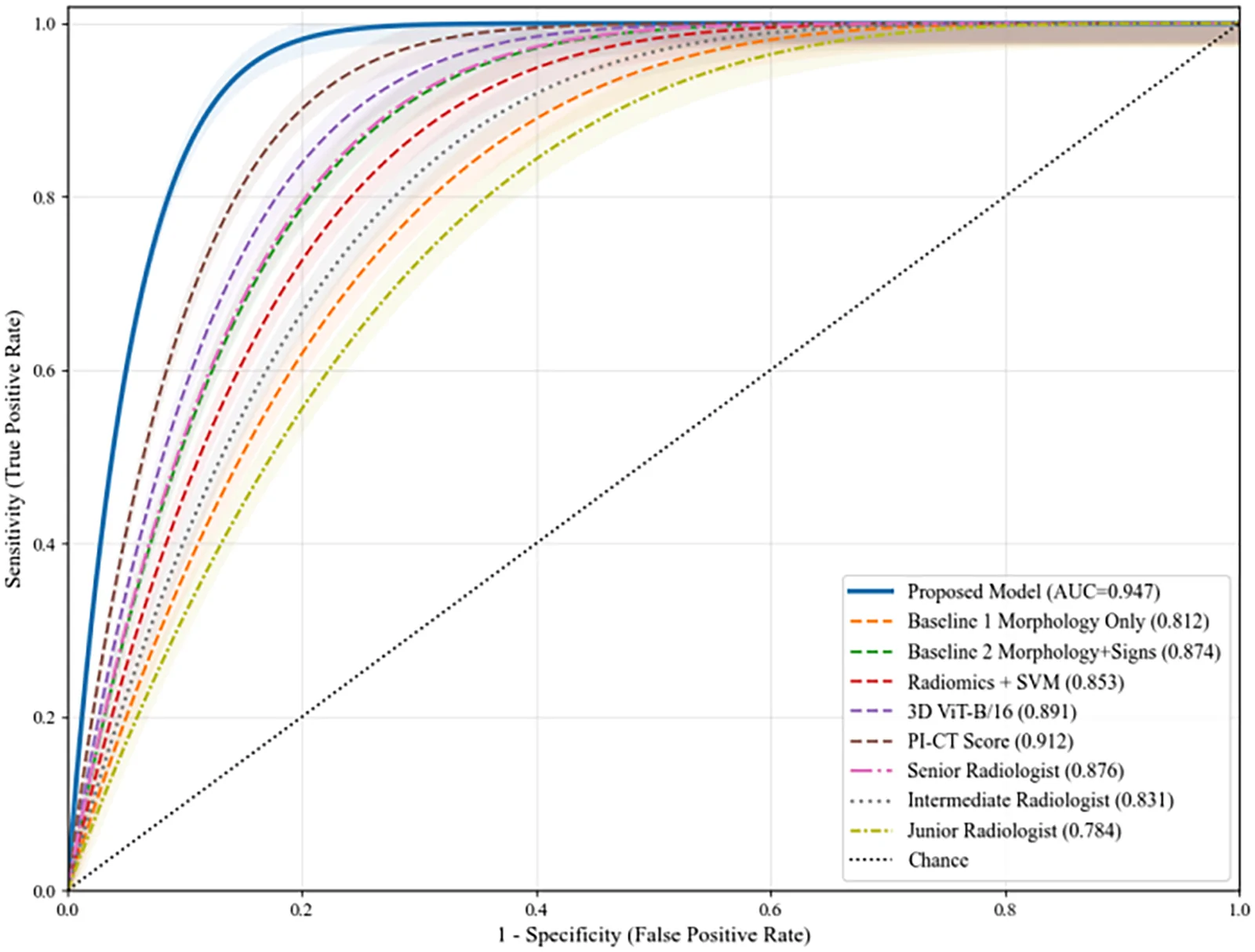
Comprehensive comparison of ROC curves of various models and doctors.

Figure 6 shows the ROC curve comparison results of the multimodal fusion CT precision diagnosis model proposed in this paper, five baseline models, and three radiologists with different levels of experience. It can be seen that the ROC curve of the model in this article is overall higher than other methods, with an AUC of 0.947, demonstrating the best diagnostic ability. In contrast, the AUC of senior, middle-aged, and junior radiologists was 0.876, 0.831, and 0.784, respectively, all lower than that of the model presented in this article. Although each baseline model can achieve a certain degree of benign and ischemic PI differentiation, its overall performance is still inferior to that of the complete model. The figure also shows the 95% confidence interval shadow bands corresponding to each curve. The results indicate that multimodal feature fusion and the cross-attention mechanism can significantly improve the recognition ability of the model for ischemic PI, achieving a better balance between sensitivity and specificity, and providing reliable support for clinical risk stratification and treatment decision-making.

To systematically and quantitatively compare the classification performance of the proposed Level 2 CT multi-feature fusion precision diagnostic model with five baseline models (including single morphology, morphology + accompanying signs, traditional radiomics + SVM, Vision Transformer, and pure PI-CT scoring system) and three radiologists with different experience levels in the differential diagnosis of benign and ischemic PI, this study presents six core indicators: sensitivity, specificity, PPV, NPV, F1 score, and Brier Score. These indicators allow for a direct evaluation of the advantages and disadvantages of each method in terms of missed diagnosis rate, false diagnosis rate, overall accuracy, and probability calibration ability, thereby verifying whether the proposed model possesses significant advantages across multiple dimensions, as shown in Table VI.

**TABLE VI.** Comprehensive comparison of classification performance indicators of each model and physician.

Model/Reader	Sensitivity (%)	Specificity (%)	PPV	NPV	F1	Brier score
Baseline 1 (Morphology only)	85.0	70.0	0.68	0.87	0.75	0.18
Baseline 2 (Morph. + Signs)	88.0	78.0	0.75	0.89	0.81	0.14
Baseline 3 (Radiomics + SVM)	82.0	76.0	0.71	0.85	0.76	0.17
Baseline 4 (ViT-B/16)	84.0	77.0	0.73	0.86	0.78	0.16
Baseline 5 (PI-CT Score)	79.0	75.0	0.69	0.84	0.74	0.19
Baseline 6 (Published score)	80.0	74.0	0.69	0.84	0.74	0.18
Proposed	91.3	93.6	0.92	0.93	0.92	0.08
Senior radiologist (10+ years)	88.0	80.0	0.76	0.90	0.82	0.13
Intermediate radiologist (5 years)	82.0	76.0	0.71	0.86	0.77	0.16
Junior radiologist (2 years)	74.0	72.0	0.65	0.80	0.69	0.21

In Table VI, the complete model presented in this paper achieved optimal values across all six metrics: sensitivity of 91.3%, specificity of 93.6%, PPV, NPV, and F1 scores of 0.92, 0.93, and 0.92, respectively, with a Brier Score as low as 0.08. In contrast, the second-best-performing model, the senior physician model (sensitivity 88.0%, specificity 80.0%, F1 = 0.82, Brier = 0.13), and the baseline 2 model (morphology + accompanying signs, sensitivity 88.0%, specificity 78.0%, F1 = 0.81), showed significant differences compared to this model. Of particular note is the fact that the specificity of the proposed model (93.6%) is significantly higher than that of senior physicians (80.0%), suggesting a significant advantage in reducing the risk of benign patients being misdiagnosed as ischemic and undergoing unnecessary surgery. Simultaneously, its Brier Score (0.08) is much lower than other comparative methods (0.13–0.21), indicating that the probability estimates output by the model are more accurate and reliable. These results collectively confirm the effectiveness of multi-feature fusion and the cross-attention mechanism in the accurate differential diagnosis of PI.

To evaluate the upstream segmentation quality that underpins the gas morphology branch, we assessed the 3D nnU-Net for intestinal wall gas segmentation on both the internal test set and the independent external validation set using Dice similarity coefficient, IoU, and Recall. These metrics reflect voxel-level overlap and the completeness of gas region detection, which directly determine the reliability of subsequent morphological feature extraction.

As shown in Table VII, the segmentation model achieved a Dice of 0.89, IoU of 0.81, and Recall of 0.91 on the internal test set, and a Dice of 0.86, IoU of 0.78, and Recall of 0.88 on the external validation set. Although the external performance decreased slightly, all metrics remained at a high level, indicating stable segmentation performance across centers and providing reliable input for the downstream gas morphology branch.

**TABLE VII.** Segmentation performance of intestinal wall gas.

Dataset	Dice	IoU	Recall
Internal test set	0.89	0.81	0.91
External validation set	0.86	0.78	0.88
Dataset	Dice	IoU	Recall

### F. Ablation experiment results

In order to further verify the effectiveness of each component module in the proposed multimodal fusion CT precision diagnosis model and its contribution to overall performance, this study conducted a systematic ablation experimental analysis. By gradually removing each module and comparing it with the baseline model, the role of each key component in predicting ischemic PI risk can be quantified, as shown in Fig. 7. At the same time, combining multiple performance evaluation indicators and module contribution analysis can help reveal the sources of model performance improvement, providing a basis for the rationality and interpretability of model design.

**FIG. 7.**
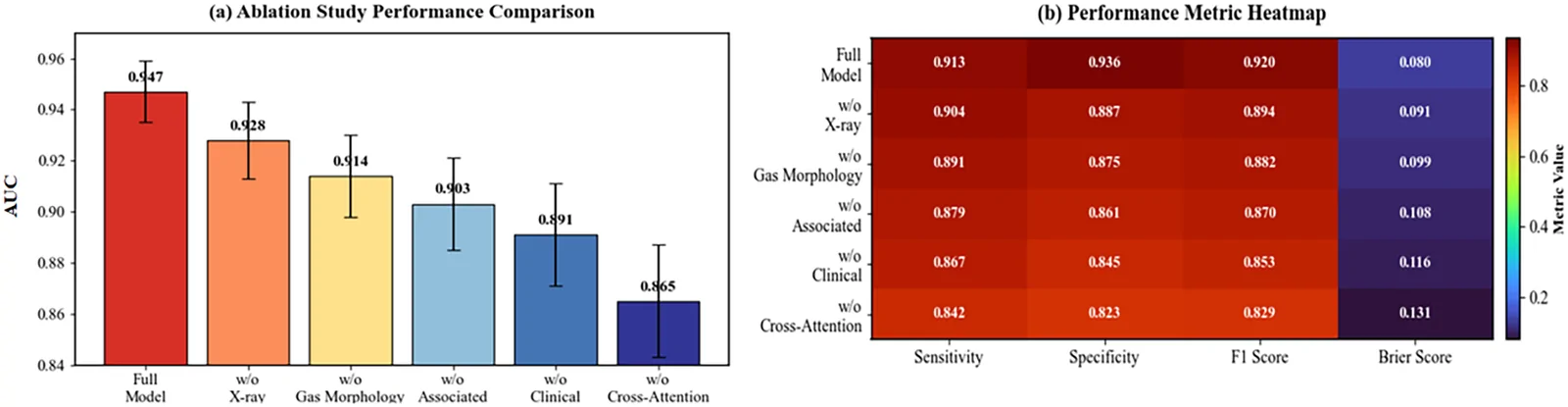
Composite diagram of contribution analysis of each module in the ablation experiment. (a) Comparison of AUC indicators after removing each module and (b) comparison of comprehensive indicators after removing each module (sensitivity, specificity, F1, Brier Score).

Figure 7 shows the experimental results of model ablation and the contribution analysis of each module. Figure 7(a) shows the comparison results of AUC for different model configurations, accompanied by 95% confidence interval error bars. It can be observed that the complete model achieved the highest AUC (0.947). However, after removing the x-ray screening level, gas morphology branch, accompanying sign branch, clinical metadata branch, or replacing the cross-attention fusion mechanism with simple feature concatenation, the performance of the model decreased to varying degrees. Among them, the performance decline is most significant after removing the cross-attention module, indicating that cross-modal feature interaction plays an important role in improving diagnostic accuracy. Figure 7(b) further illustrates the changing trends of different ablation configurations on Sensitivity, Specificity, F1 Score, and Brier Score indicators, and the complete model maintains the best performance on most indicators.

### G. Interpretability analysis results

In order to improve the interpretability of the proposed multimodal fusion CT precision diagnosis model and verify whether the model prediction results have a clear clinical basis, this study uses two complementary interpretation methods, Grad-CAM and SHAP, to analyze the decision-making process of the model. By jointly analyzing these two methods, we can further evaluate whether the model can perform risk assessment based on clinically significant information and enhance its credibility in practical clinical applications, as shown in Fig. 8.

**FIG. 8.**
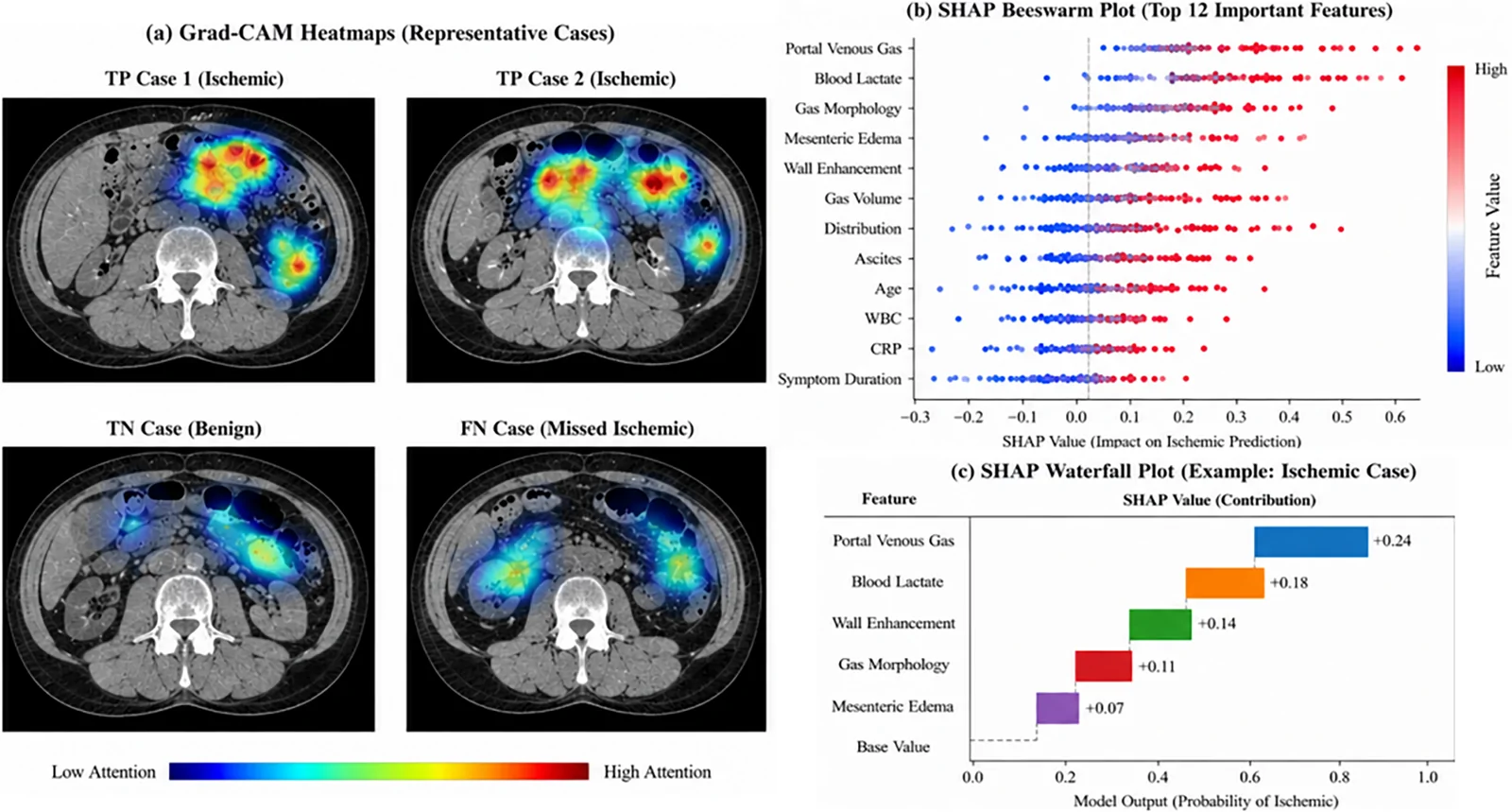
Comprehensive analysis of model interpretability. (a) Grad-CAM heatmap of representative cases; (b) SHAP swarm diagram (first 12 key features); and (c) SHAP waterfall diagram (example: ischemic case).

Figure 8(a) shows the Grad-CAM heatmap of representative cases of different case types, including 2 ischemic PI true positive cases (TP case 1, TP case 2), 1 benign PI true negative case (TN Case), and 1 ischemic PI false negative case (FN Case). The red and yellow areas in the figure represent areas of high model attention, while the blue area represents areas of low attention. Figure 8(b) shows the SHAP colony plot, which illustrates the contribution distribution of 12 important features in the test set to predicting ischemia risk. The results show that features such as Portal Venous Gas and Blood Lactate have high SHAP values, indicating that they are key factors affecting model decision-making. The high eigenvalue samples are mainly distributed in the positive SHAP region, indicating that an increase in these risk factors can significantly increase the prediction probability of the model for ischemic intestinal wall gas. Figure 8(c) shows the SHAP waterfall plot of a typical ischemic case, demonstrating the cumulative contribution process of each key feature to the final prediction result. Among them, Portal Venous Gas (+0.24) and Blood Lactate (+0.18) are the main factors driving the prediction toward the high-risk direction, while Wall Enhancement (+0.14), Gas Morphology (+0.11), and Mesenteric Edema (+0.07) further increase the model's risk assessment. These findings provide feature contribution evidence consistent with clinical knowledge; however, SHAP analysis does not establish causality or validate clinical decision-making.

### H. External validation and generalization performance

To evaluate the applicability and generalization ability of the proposed multimodal fusion CT precision diagnosis model in different clinical environments, this study further validated it using an independent external validation set (106 cases). The results showed that the model maintained high diagnostic performance on the external validation set, with an area under the ROC curve (AUC) of 0.931, sensitivity of 89.7%, and specificity of 91.2%, indicating that the model can accurately identify ischemic PI patients. Although the AUC of the external validation set has slightly decreased compared to the internal test set, the overall performance is still at a high level, indicating that the model has not shown significant overfitting and has good potential for cross-center applications.

This article further used the DeLong test to compare the differences in ROC curves between the internal test set and the external validation set, and the results showed no statistically significant difference between the two (P = 0.412). This result indicates that the diagnostic performance of the model remains stable across different data sources, and its predictive ability has good transferability and robustness. In addition, to evaluate the adaptability of the model to different devices and scanning conditions, this study conducted subgroup analysis based on CT models. The results showed that the AUC of different CT equipment subgroups ranged from 0.918 to 0.943, and the performance fluctuations between subgroups were small, with no significant device dependence observed. This indicates that the model can effectively adapt to the image differences generated by different manufacturers and models of CT equipment, and has good cross-device generalization ability.

Overall, the external validation results further demonstrate that the proposed model not only performs well on the internal test set, but also maintains stable diagnostic performance in independent datasets and different CT equipment environments, providing strong support for its future promotion and application in multicenter clinical scenarios.

## VI. CONCLUSION

This article addresses the clinical challenge of ischemic PI risk assessment by constructing a multimodal intelligent diagnostic framework that integrates gas morphological features, accompanying sign features, and clinical information, achieving accurate identification and risk stratification of ischemic PI. The results show that the model performed well on both the internal test set and the external validation set, with AUCs of 0.947 and 0.931, respectively, and demonstrated higher diagnostic accuracy and stability than radiologists of different seniority. The ablation experiment and interpretability analysis further validated the important role of the cross-attention fusion mechanism and multimodal feature collaborative modeling in performance improvement. The model focuses on regions and key feature contributions that are highly consistent with clinical cognition, demonstrating good interpretability. The research not only provides objective and reliable decision-making tools for early identification of PI ischemia risk, but also offers new technological ideas for intelligent diagnosis of complex abdominal emergencies. However, this study still has limitations, including a relatively small sample size, few external validation centers, and selection bias that may arise from the retrospective study design. The impact of first-stage false negatives on the overall diagnostic pathway requires prospective sequential evaluation. Real-world deployment requires PACS/DICOM integration, local privacy and cybersecurity approval, adequate GPU resources, and prospective workflow validation. Annotation cost is also substantial because 3D slice-by-slice segmentation is time-consuming; future work should explore semi-supervised learning, active learning, and federated learning. Future multicenter, large-sample prospective studies can incorporate more radiomics, biochemical indicators, and timely clinical data. It can also be combined with technologies such as federated learning to enhance the cross-regional generalization ability and clinical application value of the model, thereby promoting the construction and implementation of an AI-assisted PI precision diagnosis and treatment system.

## VII. METHODS

This section summarizes the core procedures for model development and validation to ensure reproducibility.

Data sources and eligibility criteria: We retrospectively collected 612 PI patients from three tertiary hospitals (North, East, and South China) between January 2022 and June 2025, and an independent external validation set of 106 cases from a fourth center (Central China). Inclusion criteria: clinically or pathologically confirmed PI; complete abdominal x-ray (erect and supine) and contrast-enhanced CT (non-contrast, arterial, portal venous phases); adequate image quality. Exclusion criteria: severe artifacts; primary intestinal tumors; >20% missing clinical data; prior major abdominal surgery. Based on final clinical diagnosis, patients were divided into benign (n = 398) and ischemic (n = 214) groups.

Image annotation: CT images were annotated slice-by-slice for intramural gas and portal venous gas (PVG) using 3D Slicer; x-ray films were labeled with bounding boxes using LabelImg. All annotations were performed by two resident radiologists (primary), reviewed by an attending radiologist, and finalized by a senior radiologist. Inter-observer agreement (Kappa) was >0.85 on 100 randomly sampled cases.

Level-1 x-ray screening model (EfficientNet-B3): Inputs were abdominal plain films pre-processed with online augmentations (brightness/contrast adjustment, Gaussian noise, slight blur, geometric transforms). The model outputs a binary probability of “PI signs visible on x ray.” Loss: weighted binary cross-entropy (positive weight = 2.0). Optimizer: Adam (lr = 3e-4) with StepLR scheduler (×0.1 every 10 epochs). Training: 100 epochs with early stopping (patience = 15). Inference time: <1 s per image.

Level-2 CT multi-feature fusion model: Preprocessing included dual window standardization (lung window had a width of 1600 HU and a level of -600 HU; abdominal window had a width of 400 HU and a level of 40 HU), resampling to 1 × 1 × 2 mm^3^ voxel spacing, and Z-score normalization. Intramural gas segmentation was performed with 3D nnU-Net (5-level encoder-decoder, deep supervision, patch size 128 × 128 × 64, Dice+Focal loss). Three parallel branches extracted features: Morphology branch: 3D ResNet-50 on lung window images guided by the segmentation mask, outputting a 2048-D vector; Sign branch: 3D RetinaNet detecting PVG, abnormal wall enhancement, mesenteric edema, and ascites, outputting a 128-D vector; Clinical branch: an MLP (two fully connected layers) processing age, lactate, WBC, log-transformed CRP, and time from symptom onset to consultation, outputting a 128-D vector. The three feature vectors were projected to 512 dimensions and fused via cross-attention (8 heads, d_k_ = 64). The fused representation passed through a fully connected classifier with Softmax to produce the ischemic PI probability. Loss: cross-entropy. Optimizer: AdamW (lr = 1 × 10^−4^, weight decay = 1 × 10^−4^) with cosine annealing (200 epochs). We trained the model on dual NVIDIA A100 80GB GPUs.

Evaluation metrics: Area under the ROC curve (AUC), sensitivity, specificity, positive and negative predictive values (PPV, NPV), F1 score, and Brier Score. We used the DeLong test to compare AUCs (α = 0.05). Interpretability was provided by Grad-CAM (3D heatmaps) and SHAP (TreeSHAP/DeepSHAP).

Baselines and ablation: Five baselines were implemented: morphology-only, morphology+signs, traditional radiomics+SVM, ViT-B/16, and the PI-CT scoring system alone. Ablation experiments sequentially removed the x-ray screening level, each branch, and the cross-attention (replaced by simple concatenation) to quantify individual contributions.

External validation: The complete pipeline was evaluated on the independent 106-case external set, with subgroup analyses by CT scanner type. We implemented all code in PyTorch 2.0. We fixed the random seed at 42 to ensure reproducibility.

## Data Availability

The data that support the findings of this study are available from the corresponding author upon reasonable request.
